# Global evolutionary dynamics of peste des petits ruminants virus reveals geographic lineage structuring and clade-defining mutational signatures

**DOI:** 10.3389/fmicb.2026.1944163

**Published:** 2026-09-30

**Authors:** Sarowar Haque, Md. Mahfuj Islam, Anupam Das, Safayet Hossain Piyel, Tanvir Ahmed, Roni Mia, Sharmin Akter, Tofazzal Islam, Md. M. N. Azim, Salima Akter, Mohammad Yaseen Ahmad, Amama Rani, Bonglee Kim, Md. Golzar Hossain

**Affiliations:** 1 Department of Microbiology and Hygiene, Bangladesh Agricultural University, Mymensingh, Bangladesh; 2 Department of Physiology, Bangladesh Agricultural University, Mymensingh, Bangladesh; 3 Institute of Biotechnology and Genetic Engineering, Gazipur Agricultural University, Gazipur, Bangladesh; 4 Department of Physiology, College of Veterinary Medicine, Jeonbuk National University, Iksan, Republic of Korea; 5 Department of Pathology, College of Korean Medicine, Kyung Hee University, Seoul, Republic of Korea; 6 Dr. B Lab Co., Ltd., Samuiwon Startup Center Kyung Hee University, Seoul, Republic of Korea; 7 Medical Convergence Research Center, Kyung Hee University Hospital, Seoul, Republic of Korea

**Keywords:** candidate phylogenetic marker, molecular evolution, PPRV, selection pressure, whole-genome sequences

## Abstract

Peste des petits ruminants virus (PPRV) is recognized as one of the most economically significant transboundary pathogens affecting small ruminants. However, despite the increasing availability of whole-genome sequences, comprehensive global analyses integrating evolutionary history, phylogeography, and genome-wide lineage-defining molecular markers remain limited. This study investigated the global genomic diversity, evolutionary history, and molecular characteristics of PPRV using whole-genome sequences. A total of 212 temporally informative whole-genome sequences, retrieved from GenBank (between 1969 and 2025) representing all four recognized PPRV lineages, were analyzed using comparative genomics, Bayesian time-scaled phylogenetic reconstruction, and genome-wide candidate phylogenetic marker; single nucleotide polymorphism (SNP) analysis and codon-based selective-pressure analyses. The results demonstrated high genomic conservation across all genomes despite their broad geographic distribution. Bayesian analysis estimated a mean evolutionary rate of 5.07 × 10^−4^ substitutions per site per year (95% HPD: 4.79 × 10^−4^ to 5.35 × 10^−4^) and dated the most recent common ancestor of PPRV to 1839 (95% HPD: 1829 to 1848) which likely reflects the ancestry of currently sampled viral diversity rather than the historical emergence of PPRV. Lineage IV, the predominant contemporary lineage, was further subdivided into four geographically structured subclades: A, B.1, B.2a, and B.2b. A total of 125 subclade-associated fixed SNP signatures were identified, primarily within the L gene, with most variants resulting in nonsynonymous substitutions. The overlapping P, V, and C coding regions underscored the complex functional consequences of lineage-defining mutations. Gene-wide dN/dS values were below 1 for all six genes, indicating predominant purifying selection, while functional mapping identified several SNP related amino acid substitutions within experimentally characterized antigenic regions; however, these substitutions were not supported as positively selected in the present analyses. These results provide a comprehensive genomic framework for understanding PPRV evolution and identify candidate phylogenetic markers for lineage discrimination to support genomic surveillance, molecular epidemiology, and the Food and Agriculture Organization (FAO)-World Organisation for Animal Health (WOAH) Global PPR Eradication Programme.

## Introduction

Peste des petits ruminants (PPR) is a highly contagious transboundary disease affecting domestic and wild small ruminants and poses a major threat to livestock production, food security, and the livelihoods of millions of resource-limited farmers across Africa, the Middle East, and Asia (WOAH, 2026). Owing to its substantial socioeconomic impact, PPR has been prioritized for global eradication by the Food and Agriculture Organization (FAO) and the World Organisation for Animal Health (WOAH) by 2030 (Kumar et al., 2017; Zhao et al., 2021).

The disease is caused by peste des petits ruminants virus (PPRV), a negative-sense single-stranded RNA virus belonging to the genus *Morbillivirus* within the family *Paramyxoviridae* (Wassif et al., 2025). The approximately 15.9-kb PPRV genome is organized in the order 3′-N-P-M-F-H-L-5′ and encodes six major proteins: nucleocapsid (N), phosphoprotein (P), matrix (M), fusion (F), hemagglutinin/attachment (H), and the large RNA-dependent RNA polymerase (L). The P gene additionally gives rise to the accessory V and C proteins through RNA editing and an overlapping open reading frame, respectively (Baron and Bataille, 2022; Kumar K. S. et al., 2014). Based on its genetic diversity, PPRV is classified into four distinct lineages (I–IV) (Alemu et al., 2019). Historically, lineages I and II have been largely confined to West Africa, whereas lineage III has predominantly circulated in East Africa and parts of the Middle East. In contrast, lineage IV has undergone remarkable geographic expansion over recent decades and is now the predominant lineage circulating throughout Asia, the Middle East, and much of Africa (Bao et al., 2025; Kwiatek et al., 2011). Advances in whole-genome sequencing have substantially enhanced genomic epidemiological investigations by providing greater phylogenetic resolution than partial gene-based analyses, thereby enabling more accurate reconstruction of viral evolution, transmission pathways, and geographic spread (Kim et al., 2025; Tiwari et al., 2025). Furthermore, Bayesian time-scaled phylogenetic approaches have facilitated the estimation of evolutionary rates, divergence times, and historical dispersal patterns, providing valuable insights into the global evolution of PPRV (Muniraju et al., 2014; Parida et al., 2015; Yang and Jeong, 2026).

Achieving the global goal of PPR eradication by 2030 requires not only effective vaccination and surveillance programmes but also a comprehensive understanding of the evolutionary dynamics, geographic dissemination, and genomic diversity of circulating PPRV populations based on complete genome sequences (Fine et al., 2020; Sarkozi, 2021). However, most previous molecular epidemiological studies of PPRV have relied on partial N- or F-gene sequences, which is useful for rapid preliminary lineage assignment while providing limited phylogenetic resolution and may not fully capture genome-wide patterns of viral evolution (Bao et al., 2017; Libeau et al., 2014; Muniraju et al., 2014; Tiwari et al., 2025). Although the number of publicly available complete PPRV genomes has increased considerably in recent years, comprehensive global studies integrating phylogeography, molecular dating, and comparative whole-genome analyses remain scarce (Kinimi et al., 2025; Philippe et al., 2011). Previous PPRV lineage and subclade classifications have relied primarily on phylogenetic clustering based on partial N/F gene sequences, with geographic distribution often providing additional epidemiological context, while more recent studies have applied whole-genome phylogenetic approaches to improve resolution (Bao et al., 2025). However, the genome-wide nucleotide substitutions consistently associated with these phylogenetically defined groups have rarely been systematically characterized (Bao et al., 2025). The identification of stable, clade-defining single-nucleotide polymorphisms (SNPs) within the studied dataset could facilitate rapid molecular classification, enhance genomic surveillance, strengthen outbreak investigations, and provide a valuable framework for monitoring viral evolution.(Cancino-Muñoz et al., 2019; Song et al., 2026; Sundermann et al., 2022). Therefore, the present study aimed to comprehensively elucidate the global evolutionary history, phylogeographic patterns, and genomic diversity of PPRV using whole genome sequences collected worldwide. In addition, we characterized genome-wide, clade-defining SNPs within the globally dominant lineage IV to provide new insights into PPRV evolution and establish robust genomic markers for future molecular surveillance, outbreak investigations, and global eradication efforts.

## Materials and methods

### Genome sequence retrieval and temporal filtering

A total of 256 whole PPRV genome sequences (minimum genome length: 15,000 bp) were retrieved from the NCBI GenBank database on May 22, 2026. Metadata associated with each sequence, including isolate/strain name, country of origin, and collection date, were compiled for subsequent analyses and isolates without collection date or country was excluded from the analysis (Supplementary file 1). All available complete PPRV genomes meeting the selection criteria were considered, irrespective of strain type or lineage. To ensure the reliability of molecular clock inference, the temporal signal of the dataset was assessed using TempEst v1.5.3 (Rambaut et al., 2016). Based on this assessment, 212 genome sequences exhibiting a strong temporal signal were retained for downstream analyses.

### Geographic distribution and pairwise nucleotide identity analysis

The geographic distribution and country-level prevalence of PPRV lineages were analyzed and visualized using Python with the Pandas, NumPy, Matplotlib, and GeoPandas libraries. Dominant lineages were identified based on sequence frequency and displayed on a global choropleth map. The filtered genome sequences were aligned using MAFFT v7.505 (Katoh and Standley, 2013) and subsequently inspected, manually refined, and trimmed using Unipro UGENE v53.1 (Okonechnikov et al., 2012). Pairwise nucleotide identity analysis was performed using custom Python scripts implemented with the Biopython, NumPy, Pandas, Seaborn, and Matplotlib libraries. Pairwise nucleotide identities among the 212 aligned complete genomes were calculated after excluding gaps and ambiguous nucleotide positions. The resulting identity matrix was visualized as a hierarchically clustered heatmap using average-linkage clustering based on pairwise nucleotide distances. Lineage annotation bars were added to facilitate interpretation.

### Time-resolved phylogenetic analysis

Time-resolved phylogenetic reconstruction was performed using Bayesian Evolutionary Analysis Sampling Trees (BEAST) v1.10.5 with the aligned whole-genome sequences (Baele et al., 2025). The best-fitting nucleotide substitution model was determined using jModelTest v2.1.10 (Darriba et al., 2012) based on the Akaike Information Criterion (AIC), which identified the GTR + I + G model as the optimal substitution model. Strict and uncorrelated lognormal relaxed molecular clock models were compared using marginal likelihood estimation based on path-sampling and stepping-stone sampling under a constant population-size coalescent prior. For both clock models, marginal likelihood analyses were run for 50 path steps, with 2 million generations per step. Marginal likelihood estimates supported the use of uncorrelated relaxed clock model under coalescent constant size population for the final Bayesian analysis. However, several relaxed-clock-specific parameters showed insufficient sampling and poor mixing despite increasing chain length (400 M), particularly parameters associated with among-lineage rate variation. Therefore, because reliable estimation of branch-specific evolutionary rates could not be achieved under the relaxed-clock framework, the strict molecular clock model was selected for downstream Bayesian inference based on its stable convergence and satisfactory parameter sampling. The final Markov chain Monte Carlo (MCMC) analysis was run for 100 million generations, with sampling performed every 10,000 generations. Convergence and mixing of the MCMC chains were assessed using Tracer v1.7.2 (Rambaut et al., 2018). All parameters achieved effective sample size (ESS) values greater than 200 after discarding the initial 10% of samples as burn-in, indicating adequate sampling and convergence Community B (2025). A maximum clade credibility (MCC) tree was generated using TreeAnnotator with median node heights and subsequently visualized using FigTree v1.4.4 Figtree (2007). Within lineage IV, phylogenetic groups were designated according to the hierarchical topology of the whole-genome MCC tree for descriptive and comparative purposes. The major lineage IV split separated clade A from a broader clade B. Clade B was subsequently divided into B.1 and B.2, with B.2 further separating into B.2a and B.2b.

### Clade-defining SNP analysis

Clade-defining SNPs among lineage IV PPRV genomes were identified using custom Python scripts following multiple sequence alignment of all lineage IV whole-genome sequences (152/212) against the reference genome of lineage IV NC_006383.2 (Muniraju et al., 2014). A nucleotide position was defined as clade-defining when all sequences within a given subclade shared the same nucleotide, while the remaining subclades possessed an alternative conserved nucleotide at that position. The identified SNPs were compiled into a clade-defining nucleotide matrix. Genomic coordinates of all SNPs were mapped onto the reference genome, and coding sequence annotations were extracted from the corresponding GenBank file. Each SNP was assigned to its respective coding or intergenic region, and codon changes were translated to determine the associated amino acid substitutions. Based on their predicted effects, mutations were classified as synonymous, nonsynonymous, or intergenic. Custom Python scripts utilizing Biopython, Pandas, NumPy, and Matplotlib were employed to generate the clade-defining SNP matrix, visualize the genomic distribution and functional effects of SNPs across the PPRV genome, and illustrate the overlapping genomic organization of the P, V, and C open reading frames. To evaluate the reliability of the identified SNP signatures, additional validation analyses were performed using custom python scripts. Potential homoplastic substitutions were assessed across all clade-defining SNP positions. A concordance threshold analysis was conducted to determine the minimum proportion of matching SNPs required for subclade assignment. Leave-one-out cross-validation (LOOCV) was performed as an internal robustness assessment by evaluating the consistency of SNP-based subclade classification.

### N- and F-gene phylogenetic analysis and subclade concordance

Complete N- and F-gene coding sequences from the 152 lineage IV genomes were analyzed separately using IQ-TREE v2.0.7 (Nguyen et al., 2015). ModelFinder implemented in IQ-TREE selected K3P + G4 for the N gene and TIM2e + G4 for the F gene based on BIC. Branch support was assessed using 1,000 SH-aLRT and 1,000 ultrafast bootstrap replicates. Concordance with the whole-genome-defined subclades was evaluated using exact clade recovery, precision, recall, F1 score, and Jaccard similarity. Clade-defining SNPs within the N and F genes were also compared with the whole-genome subclade assignments.

### Codon-based selection pressure analysis of lineage IV

Selection pressure acting on the six major PPRV protein-coding genes (N, P, M, F, H, and L) was evaluated using HyPhy v2.5 (Hypothesis Testing Using Phylogenies) (Pond et al., 2005). Coding sequences from the 152 lineage IV genomes were aligned separately for each gene while maintaining the correct codon reading frame, and the corresponding lineage IV phylogenetic tree was used for the analyses. Site-specific selection across lineage IV was assessed using three complementary methods: Fixed Effects Likelihood (FEL), Fast Unconstrained Bayesian AppRoximation (FUBAR), and Mixed Effects Model of Evolution (MEME). FEL was used to identify codons showing evidence of pervasive positive or negative selection, with *p* ≤ 0.10 used as the candidate threshold. FUBAR was used to identify codons under pervasive diversifying selection, with posterior probability ≥ 0.90 considered supportive. MEME was used to detect episodic diversifying selection acting on a subset of phylogenetic branches, with *p* ≤ 0.10 used as the candidate threshold. To strengthen site-level inference, codons detected by at least two of the three methods were considered to have stronger cross-method support, whereas sites identified simultaneously by FEL, FUBAR, and MEME were regarded as the strongest candidates for diversifying selection.

To examine subclade-defining selection patterns, FEL was additionally applied separately to subclades A, B.1, B.2a, and B.2b. Formal differences in codon-specific selective pressure among the better-sampled B.1, B.2a, and B.2b subclades were subsequently evaluated using Contrast-FEL, while subclade A was retained as background in the phylogeny. Synonymous-rate variation and permutation testing were enabled. Nominal significance was assessed at *p* ≤ 0.05, and false-discovery-rate correction was applied to account for multiple codon-level comparisons, with *q* ≤ 0.20 used to identify robust differences in selective pressure among subclades. Also, nonsynonymous SNPs were compared with codons identified by the selection analyses to assess whether subclade-defining amino-acid substitutions coincided with sites showing evidence of diversifying selection.

## Results

### Global distribution of PPRV lineages and genomic divergence

A total of 256 whole PPRV genome sequences deposited in GenBank between 1969 and 2025 were identified and classified into four distinct lineages (I–IV). Following temporal signal assessment, 212 whole genome sequences were retained for subsequent analyses. The dataset revealed that lineage IV was the predominant lineage worldwide, accounting for 71.70% (152/212) of all sequences, followed by lineage II (20.28%, 43/212), lineage III (5.66%, 12/212), and lineage I (2.36%, 5/212) (Figure 1). Lineage IV exhibited the broadest geographical distribution, spanning Asia, the Middle East, and Africa. China contributed the largest number of lineage IV genomes (*n* = 69), followed by India (*n* = 28) and Israel (*n* = 19). In contrast, lineage II was primarily restricted to West Africa, with the highest numbers reported from Mali (*n* = 14) and Senegal (*n* = 12) (Figure 1). Lineage III was detected across several East African countries, including Ethiopia, Uganda, Tanzania, Kenya, Sudan, and Burundi. Conversely, lineage I remained confined to West Africa and was represented by only five whole genome sequences (Figure 1). However, this distribution reflects available genomic sampling rather than necessarily representing the absolute global prevalence of each lineage. Pairwise nucleotide identity analysis demonstrated substantial genomic diversity among global PPRV isolates, with overall nucleotide identities ranging from 86.17 to 99.99% (Figure 2). The highest nucleotide identity (99.99%) was observed between two Chinese lineage IV isolates (MF443343.1 and MF443348.1) collected in 2014. In contrast, the lowest nucleotide identity (86.17%) was detected between a Romania lineage IV isolate collected in 2024 (PV385050.1) and a Burundi lineage III isolate collected in 2017 (MK686066.1) (Figure 2, Supplementary file 2). Collectively, these findings indicate that lineage IV is the globally dominant and most geographically widespread PPRV lineage, while the virus maintains considerable genomic diversity across its global distribution.

**Figure 1:**
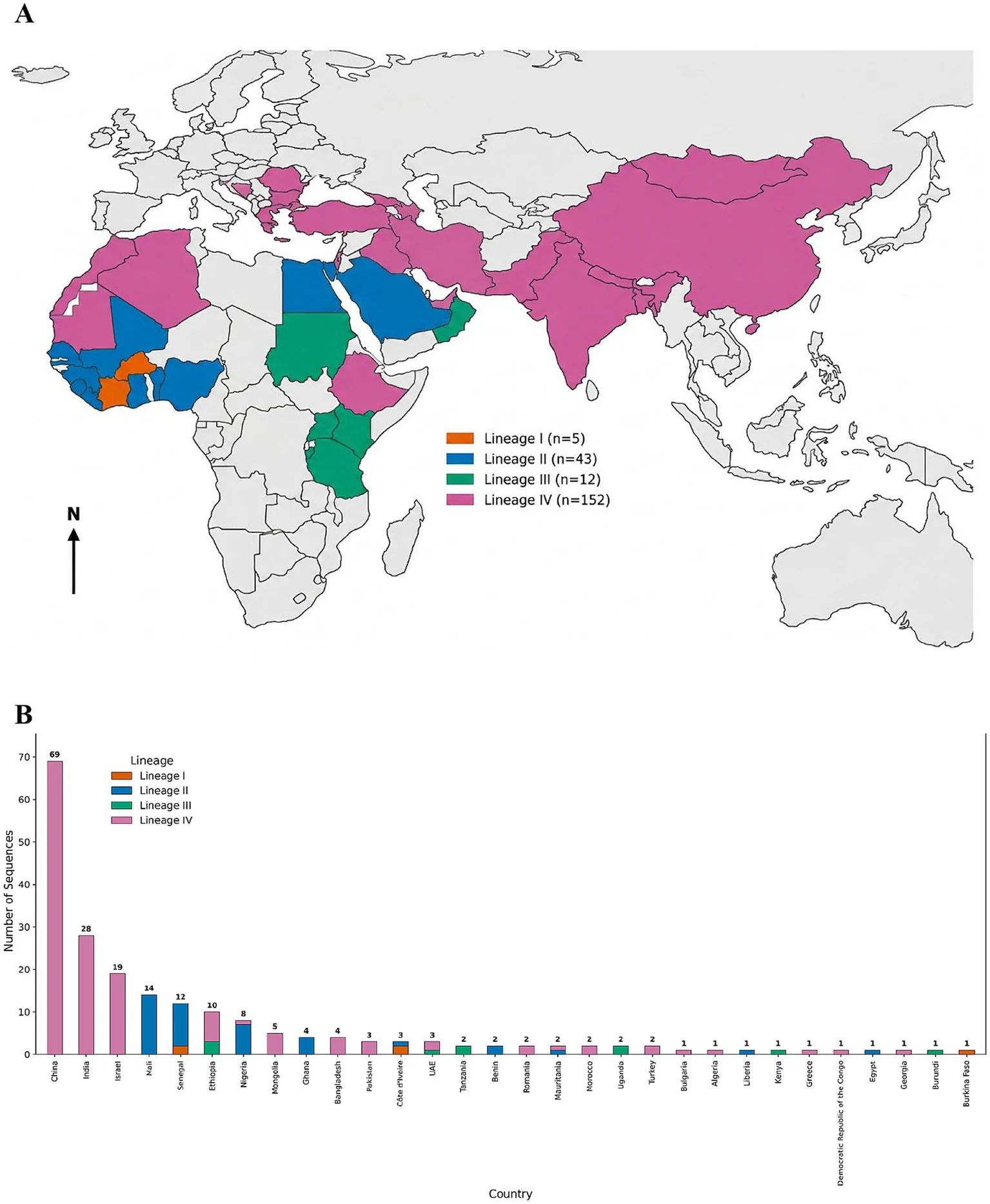
Geographical distribution of global PPRV lineages. Metadata from 212 whole PPRV genome sequences, including country of origin and lineage classification, were compiled in Microsoft Excel. Data processing, analysis, and visualization were performed in Python using the Pandas, NumPy, Matplotlib, and GeoPandas libraries. Colors represent the corresponding PPRV lineages. **(A)** Global geographic distribution of PPRV lineages based on country of origin. **(B)** Country-wise frequency distribution of PPRV lineages.

**Figure 2:**
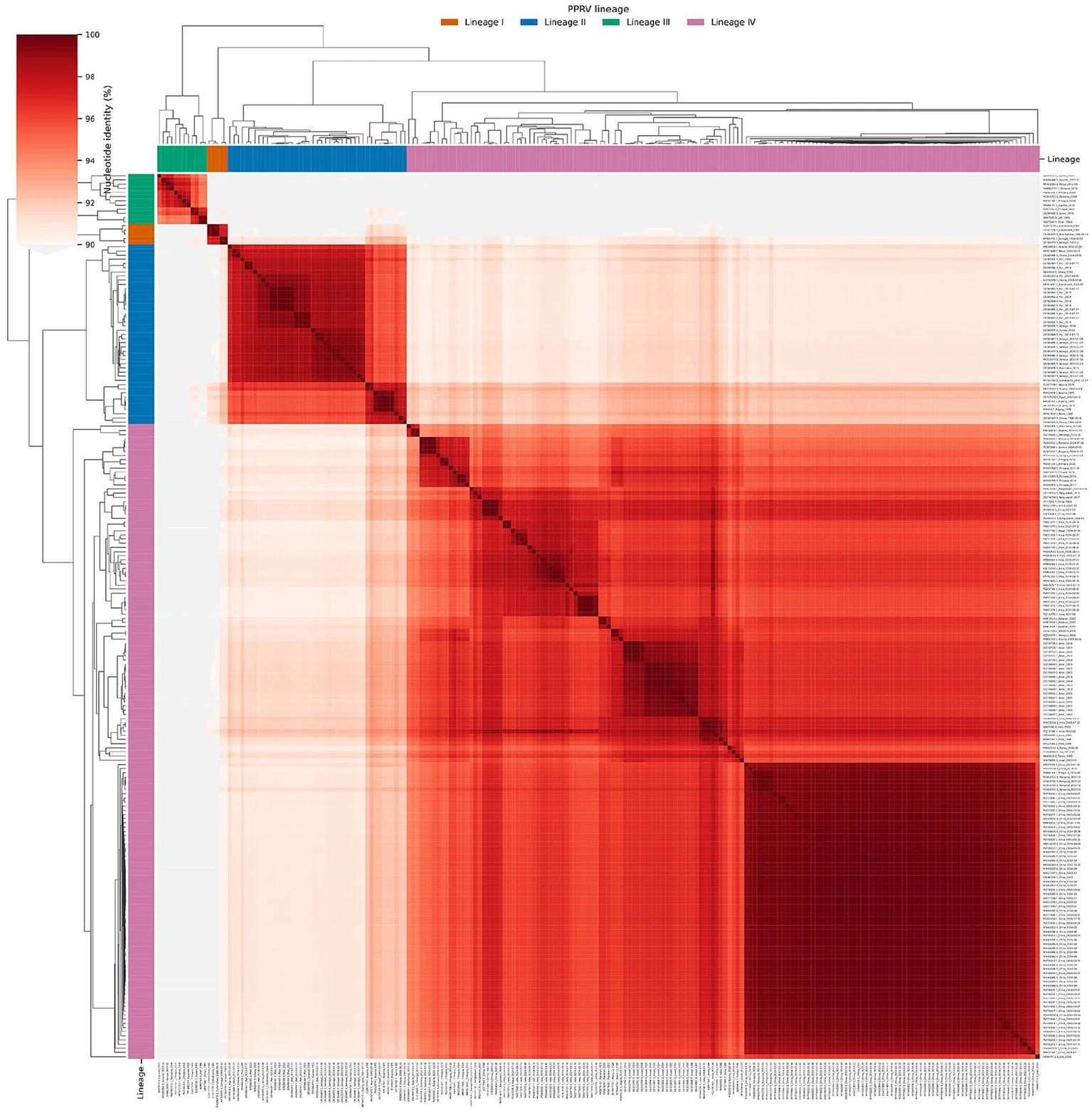
Pairwise identity matrix of global PPRV genomes. Pairwise nucleotide identities were calculated after excluding gaps and ambiguous nucleotide positions. Hierarchical clustering was performed using average linkage based on pairwise nucleotide distances. Row and column annotation bars indicate PPRV lineage membership. The heatmap color scale is displayed from 90% to 100% identity; values below 90% are represented at the lower limit of the scale.

### Temporal evolution and phylogeography of PPRV

Root-to-tip regression analysis was performed to assess the temporal signal among the 212 global PPRV genome sequences. A strong positive correlation was observed between genetic divergence and sampling dates, with a correlation coefficient of 0.8991 and a coefficient of determination (*R*^2^) of 0.8084 (Supplementary Figure S1). Bayesian molecular clock analysis estimated a mean evolutionary rate of 5.07 × 10^−4^ nucleotide substitutions per site per year (95% HPD: 4.79 × 10^−4^–5.35 × 10^−4^ substitutions/site/year) (Supplementary Figure S2). The time to the most recent common ancestor (TMRCA) of all sampled PPRV sequences was estimated to be 1839, with a 95% HPD interval ranging from 1829 to 1848 (Figure 3A). The time-resolved phylogeny revealed four well-supported monophyletic lineages, with strong posterior probabilities supporting major clades and subclades. Lineage III exhibited the oldest estimated TMRCA (1943; 95% HPD: 1939–1947), followed by lineages II, I, and IV (Figure 3A, Table 1). Lineage II was divided into two major clades. Clade A (TMRCA: 1956; 95% HPD: 1954–1958) contained a single geographically distinct North African isolate from Egypt (1992), whereas both clades A and B (TMRCA: 1960; 95% HPD: 1958–1963) were predominantly composed of West African isolates from Benin (1969–2011), Nigeria (1975–2012), Ghana (1986–2014), Mali (1999–2016), Senegal (2010–2016), Mauritania (2012), Guinea (2013), and Liberia (2015) (Figure 3B). Lineage I, represented by only five genomes, was exclusively confined to West Africa and comprised historical viral strains circulating in Senegal (1969–1995), Burkina Faso (1986), and Côte d’Ivoire (1989). In contrast, lineage III consisted of two subclades. Subclade III-A contained only two sequences originating from Oman and the United Arab Emirates, whereas subclade III-B was restricted to East Africa and included isolates from Sudan (1972), Ethiopia (1994–2019), Kenya (2011), Uganda (2012–2020), Tanzania (2016–2018), and Burundi (2017) (Figure 3B, Table 1). Lineage IV, the most prevalent lineage globally, had an estimated TMRCA of 1954 (95% HPD: 1950–1957).

**Figure 3:**
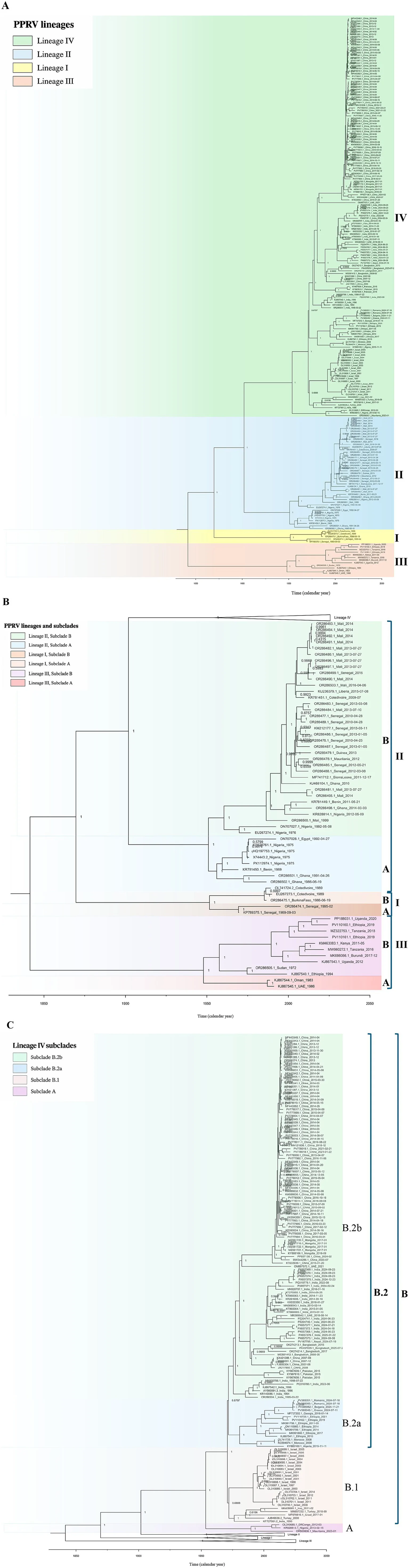
Time-resolved phylogenetic tree of complete PPRV genomes. A total of 212 complete PPRV genomes were retrieved from GenBank, and time-resolved phylogenetic trees were inferred using Bayesian Markov chain Monte Carlo (MCMC) analysis. The trees were estimated under the GTR + I + G nucleotide substitution model with a strict molecular clock and a coalescent constant-population-size tree prior. The x-axis represents calendar years corresponding to sampling dates and estimated ancestral divergence times, and posterior probabilities are shown at each major nodes. **(A)** Phylogenetic tree showing the four PPRV lineages. **(B)** Subclades of lineages I, II, and III. **(C)** Subclades of lineage IV.

**Table 1:** Estimated TMRCA and 95% HPD intervals for PPRV lineages and major subclades.

Lineage/clade	Median node height (years)	TMRCA (year)	95% HPD (year)	Number of genomes (*n*)
Lineage I	74.03	1952	1948–1955	5
I-A	56.15	1969	1969–1970	2
I-B	40.38	1985	1985–1986	3
Lineage II	74.65	1951	1948–1953	43
II-A	69.57	1956	1954–1958	8
II-B	65.17	1960	1958–1963	35
Lineage III	82.55	1943	1939–1947	12
III-A	43.37	1982	1981–1983	2
III-B	72.80	1953	1949–1956	10
Lineage IV	72.04	1954	1950–1957	152
IV-A	35.84	1990	1987–1992	3
IV-B	54.66	1971	1969–1973	149
IV-B.1	53.51	1972	1970–1974	23
IV-B.2	53.86	1972	1969–1974	126
IV-B.2a	44.90	1981	1978–1983	15
IV-B.2b	49.90	1976	1973–1978	111
Overall PPRV	186.56	1839	1829–1848	212

The whole-genome phylogeny revealed a hierarchical structure within lineage IV. The major split separated clade A from clade B; clade B subsequently separated into B.1 and B.2, while B.2 further comprised B.2a and B.2b, and the TMRCA and corresponding 95% HPD intervals were estimated for each subgroup (Figure 3C, Table 1). The largest subclade, B.2b, demonstrated sustained evolutionary connectivity across East and South Asia. This subclade was dominated by isolates from China (2007–2024) and India (1994–2025), with additional sequences from Bangladesh (2008–2025), Pakistan (2015), Mongolia (2016–2017), Nepal (2024), and the United Arab Emirates (2018–2021). Subclade B.2a comprised isolates from Morocco (2008), Ethiopia (2010–2021), Algeria (2015), and Georgia (2016), and also included recent detections in southeastern Europe, namely Greece, Romania, and Bulgaria (2024). In contrast, subclade B.1 was predominantly represented by isolates from Israel, where the virus persisted for approximately two decades (1997–2017), together with isolates from Turkey (2000–2018) and Iraq (2011). Notably, a single Indian isolate collected in 1996 clustered within this subclade (Figure 3C). Taken together, these findings demonstrate that PPRV has diversified into four geographically structured lineages, with lineage IV exhibiting the greatest genetic diversification, long-term persistence, and transcontinental dissemination during recent decades.

### Clade-defining SNP landscape of lineage IV

Comparative SNP analysis using the reference genome NC_006383.2 identified 125 unique clade-defining SNPs distributed across nucleotide positions 513–14710 that distinguished the four major subclades within lineage IV (Figure 4A). Subclade A harbored the largest number of lineage-defining SNPs (*n* = 115), whereas subclade B.2a was characterized by eight SNPs. In contrast, subclades B.1 and B.2b were each defined by a single substitution located at nucleotide positions 2,540 and 3,555, respectively (Figure 4A; Supplementary file 3). Gene-wise distribution analysis revealed that the L gene contained the highest number of SNPs (*n* = 45), followed by the H (*n* = 21), F (*n* = 19), N (*n* = 9), P (*n* = 9), and M (*n* = 7) genes. Notably, the nine clade-defining SNPs identified within the P gene also affected the V and C proteins because of the overlapping genomic organization of these genes (Figures 4B,C). These mutations comprised both synonymous and nonsynonymous substitutions (Figure 4D). These findings indicate that the diversification of lineage IV is characterized by subclade-defining SNP signatures, with the majority of lineage-defining mutations accumulating within the L gene, highlighting its substantial contribution to the evolutionary divergence of this lineage. Homoplasy assessment identified no potential homoplastic sites among the 125 clade-defining SNP positions. Threshold analysis showed that a concordance threshold of minimum 0.51 eliminated competing-clade assignments while maintaining a 98.68% correct assignment rate within the evaluated dataset. Leave-one-out cross-validation demonstrated high assignment performance across lineage IV subclades, with sensitivity ranging from 99.1% to 100%, specificity of 100%, and F1 scores ranging from 0.995 to 1.000 (Supplementary Table S1). These results indicate robustness within the analyzed dataset without representing independent external validation.

**Figure 4:**
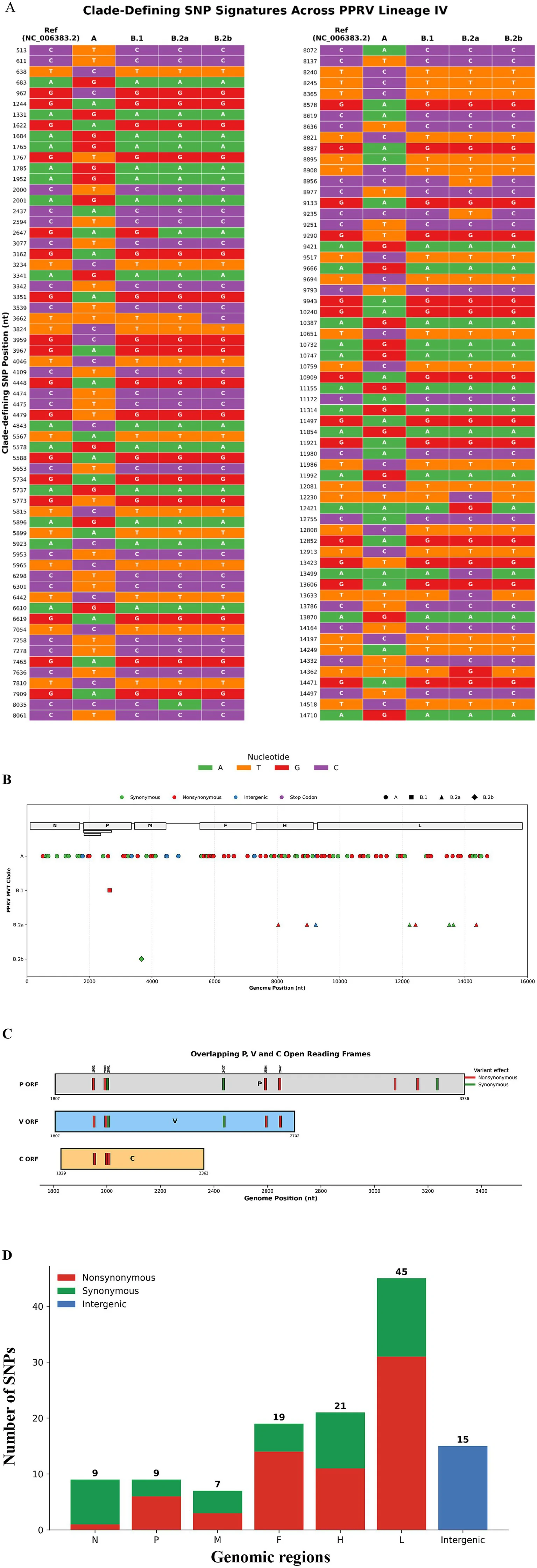
Clade-defining SNP characterization of lineage IV PPRV subclades. Clade-defining SNP identification, annotation, and visualization were performed for 152 complete lineage IV PPRV genomes using custom Python scripts. **(A)** Clade-defining SNP matrix showing 125 SNPs and their nucleotide positions distinguishing lineage IV subclades. **(B)** Functional classification and visualization of clade-defining SNPs. **(C)** Schematic representation of overlapping coding regions and the predicted functional effects of clade-defining SNPs. **(D)** Gene-wise distribution of clade-defining SNPs across the PPRV coding regions.

Among the nonsynonymous SNPs, nucleotide (nt) 7636 C > T produced the H-protein substitution G104V within the previously characterized linear B-cell epitope at residues 98–107 (Yu et al., 2017). In addition, H271L and E347A mapped within the antibody-binding region at residues 263–368, while D544V and Y551F mapped within the 538–609 region. Additionally, both N- and F-gene phylogenies showed high concordance with the whole-genome lineage IV subclades. The N-gene tree exactly recovered A, B.1, and B.2a, with an overall macro-F1 of 0.989, whereas the F-gene tree exactly recovered A and B.2a, with a macro-F1 of 0.988. Concordance for B.1 and B.2b was high but incomplete. Nine SNPs were located in the N gene and 19 in the F gene, all associated with subclade A. Thus, N/F-region SNPs distinguished subclade A from the B-group subclades but did not independently discriminate B.1, B.2a, and B.2b (Supplementary Table S2).

### Selection pressure landscape of lineage IV

Gene-wide analyses indicated that all six major PPRV genes were predominantly under purifying selection, with dN/dS values below 1 for N (0.1446), *p* (0.4856), M (0.0818), *F* (0.1282), H (0.2064), and L (0.1207) (Supplementary file 4). Despite predominant gene-wide purifying selection, site-specific analyses identified 18 individual codons with cross-method evidence of localized diversifying selection, each supported by at least two of FEL, FUBAR, and MEME. Of these, nine codons were supported by all three methods: N479; P77, P83, P139, P199, P284, and P421; and L641 and L1257 (Figure 5). Formal Contrast-FEL analysis detected no significant differences in codon-specific selective pressure among B.1, B.2a, and B.2b after FDR correction (*q* ≤ 0.20). Although N441, P43, and L319 showed nominal omnibus significance, none remained significant after multiple-testing correction. Thus, the subclade-defining Contrast-FEL analysis provided no robust evidence of differential selective pressure among the three B subclades. Comparison of nonsynonymous clade-defining substitutions with the selection-supported codons showed only limited overlap. F-protein A18G substitution (codon 18) coincided with a whole-lineage IV selection signal, and this was detected by MEME alone, whereas none of the substitutions overlapped codons supported by at least two selection methods (Supplementary file 5).

**Figure 5:**
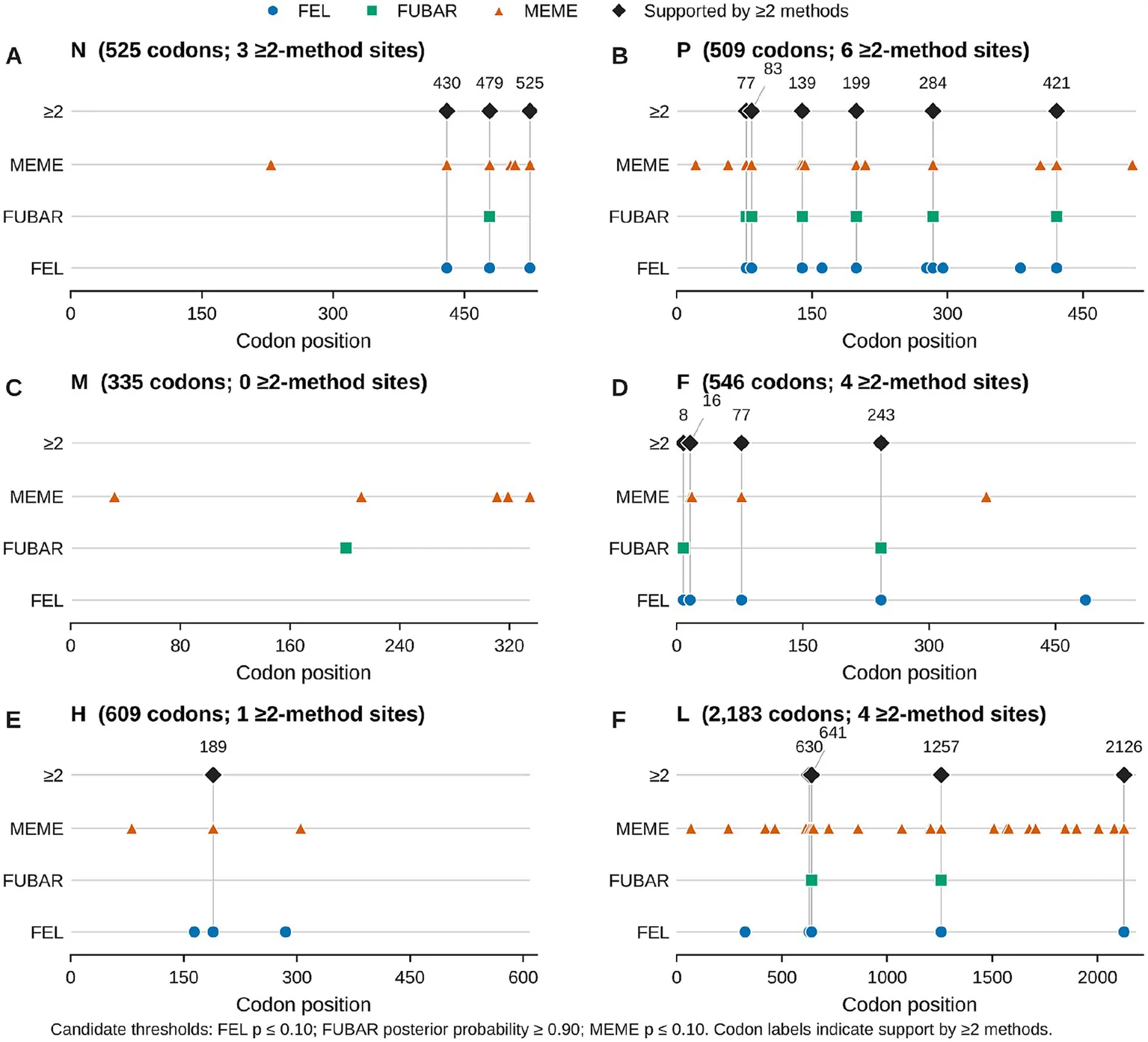
Selection pressure landscape of lineage IV PPRV proteins. Site-specific selection was assessed using FEL, FUBAR, and MEME across 152 lineage IV genomes. **(A–F)** Selection-supported codons across N, P, M, F, H, and L proteins.

## Discussion

PPRV remains a major threat to global small-ruminant health despite the availability of an effective vaccine and the ongoing eradication initiatives led by the FAO and WOAH (Kumar et al., 2017; Zhao et al., 2021). However, persistent circulation is associated with challenges in achieving sufficient vaccination coverage, particularly among smallholder and mobile pastoral livestock populations, limitations in surveillance and early detection, unequal access to vaccine delivery systems, and continued virus dissemination through livestock movement and cross-border transmission (Acosta et al., 2022; Afari-Asiedu et al., 2024). Therefore, understanding the evolutionary dynamics and geographic spread of PPRV in endemic regions is critical for strengthening surveillance systems, tracking transmission pathways, and informing evidence-based control and eradication strategies (Imanbayeva et al., 2025).

In the present study, we investigated the global evolutionary patterns of PPRV genomes collected between 1969 and 2025 using an integrated framework combining phylogenetic reconstruction, comparative genomic analysis, clade-defining SNP characterization, and codon-based selection-pressure assessment. The findings confirm that PPRV lineage IV predominates across Asia, the Middle East, and Africa, consistent with previous reports indicating that this lineage has progressively displaced the historically circulating African lineages (Kwiatek et al., 2011; Mantip et al., 2019; Muniraju et al., 2014). The most plausible explanation for this expansion is epidemiological rather than evolutionary, driven by factors such as extensive transboundary livestock movement, informal animal trade, and repeated introductions into susceptible sheep and goat populations, rather than by an increased evolutionary rate of the virus itself (Fèvre et al., 2006; Hui, 2006; Legnardi et al., 2022; Wang et al., 2009). In contrast, the continued confinement of lineages I and II to West Africa and lineage III to East Africa likely reflects historical transmission networks and regional animal movement patterns that have maintained geographically structured viral populations despite decades of circulation (Adombi et al., 2017; Kwiatek et al., 2011; Tounkara et al., 2021).

The global PPRV population exhibited remarkably high nucleotide conservation, with an estimated evolutionary rate of 5.07 × 10^−4^ substitutions per site per year. This finding, supported by both Bayesian molecular clock and pairwise identity analyses, is consistent with the relatively conserved evolutionary characteristics of morbilliviruses (Barry et al., 2024; Beaty and Lee, 2016). These observations suggest that PPRV evolution is driven primarily by the gradual accumulation and fixation of point mutations rather than by rapid genome diversification (Barry et al., 2024; Padhi and Ma, 2014). Consequently, despite its continuous geographic expansion, the evolutionary dynamics of PPRV appear to have remained relatively stable over time (Bao et al., 2025; Mahapatra et al., 2021; Padhi and Ma, 2014). Bayesian molecular dating suggests that the current genetic diversity of PPRV emerged during the early nineteenth century, supporting previous estimates that viral diversification occurred long before the disease was formally recognized (Mahapatra et al., 2021). However, it may not be interpreted as indicating that PPR as a disease emerged during this period. Rather, the estimated TMRCA represents the common ancestor of the currently available genomic dataset and is strongly influenced by the temporal and geographic sampling structure of available sequences. Although lineage III is widely considered the oldest extant lineage (Muniraju et al., 2014), the estimated time to the most recent common ancestor (TMRCA) reflects only the ancestry of currently sampled viruses and should not be interpreted as the absolute origin of PPRV, since extinct or unsampled lineages are not represented in phylogenetic reconstructions (Wertheim and Kosakovsky Pond, 2011). The approximately century-long interval between the overall PPRV TMRCA and the emergence of currently sampled lineage-level diversity likely represents an unsampled ancestral period rather than an absence of viral circulation (Muniraju et al., 2014; Padhi and Ma, 2014).

Despite the global predominance of lineage IV, its subclades exhibit distinct geographic structuring. Subclade B.2b predominates throughout East and South Asia, subclade B.1 remains largely restricted to Israel, Turkey, and Iraq, whereas subclade B.2a circulates in North and East Africa and has recently been detected in southeastern Europe. This distribution pattern suggests continued transboundary dissemination through livestock movement and trade (Padhi and Ma, 2014). However, these findings may be interpreted cautiously because of uneven genomic sampling across both lineages and geographic regions. The overrepresentation of lineage IV and genomes from countries such as China and India may influence lineage-specific evolutionary estimates and geographic patterns. Thus, the observed patterns likely reflect the available genomic sampling rather than the true global diversity and distribution of PPRV.

The heterogeneous distribution of clade-defining SNPs across viral genes highlights the varying evolutionary constraints acting on different regions of the PPRV genome. The L gene contained the highest number of the variants, which is likely attributable to its large coding capacity and its essential role in encoding the viral RNA-dependent RNA polymerase responsible for genome replication and transcription (Diallo et al., 2002; Prajapati et al., 2019; Rajan et al., 2025). Similarly, the H and F genes, which encode the attachment and fusion glycoproteins involved in receptor recognition and membrane fusion, respectively, also harbored substantial numbers of lineage-defining substitutions (Prajapati et al., 2019). The accumulation of lineage-specific mutations within these genes may reflect a balance between long-term viral adaptation and functional constraints imposed on proteins that play critical roles in viral entry and host interaction. However, the biological significance of these amino acid substitutions remains unknown and requires experimental validation to determine their potential effects on viral replication, transmission efficiency, virulence, and antigenic properties (Baron et al., 2016). Notably, more than half of the identified clade-defining variants were nonsynonymous, suggesting that lineage diversification has involved alterations in protein-coding sequences in addition to the accumulation of silent mutations. Although synonymous substitutions are generally considered selectively neutral, they remain valuable molecular markers for reconstructing evolutionary histories and defining phylogenetic relationships (Martinez et al., 2025; Zhang and Qian, 2025). Furthermore, the clade-defining variants identified within the overlapping P, V, and C coding regions illustrate the evolutionary complexity of the PPRV genome, where a single nucleotide substitution may simultaneously affect multiple gene products. Such genomic organization imposes additional evolutionary constraints and underscores the importance of interpreting mutations within their broader genetic context (Kumar N. et al., 2014). The strong consistency between SNP-based assignments and whole-genome-defined classifications suggests that these genomic signatures capture the underlying evolutionary structure of the analyzed viruses (Li et al., 2026). However, it is worth noting that these SNP signatures may complement conventional phylogenetic approaches by improving discrimination among closely related viral clades. Further validation across broader temporal and geographic datasets is required before their routine diagnostic application (Hadfield et al., 2018). In contrast, the identified SNP signatures showed high internal consistency, their performance on independent surveillance datasets remains unknown. Therefore, these signatures might currently be considered candidate phylogenetic markers rather than validated diagnostic classifiers or standalone molecular typing tools. Notably, a limitation of the SNP analysis is the unequal representation of lineage IV subclades, particularly subclade A, which comprises only three genomes. Although the identified subclade A-associated positions were conserved among the available genomes, additional sequences will be required to determine whether these signatures represent broadly conserved lineage characteristics or dataset-specific patterns. The high concordance of N- and F-gene phylogenies with the whole-genome classification supports their use for preliminary molecular classification within the studied dataset. However, incomplete resolution of some B subclades and the limited discriminatory value of N/F-region SNPs indicate that whole-genome analysis provides greater resolution among closely related lineage IV subclades suggesting two approaches are complementary (Pearson et al., 2009).

PPRV coding regions were dominated by purifying selection, while a limited number of codons showed localized diversifying-selection signals which aligns with the previous studies of PPRV (Courcelle et al., 2024; Shabbir et al., 2020). This might be because of gene-wide dN/dS represents the average selective pressure across all codons, whereas FEL, FUBAR, and MEME detect site-specific selection. Thus, the findings indicate localized selective variation within an overall background of strong evolutionary constraint rather than widespread adaptive evolution (Kosakovsky Pond et al., 2021). Importantly, the formal Contrast-FEL analysis was negative after correction for multiple testing, with no codon showing significant differential selective pressure among B.1, B.2a, and B.2b. Therefore, the present data may not support robust subclade-defining differences in selective pressure, despite several nominal site-level signals. Interpretation of subclade A was particularly limited because only three genomes were available, reducing the power to detect subclade-defining selection signals.

In conclusion, these findings demonstrate that PPRV has evolved into four geographically structured lineages, with lineage IV emerging as the globally predominant and most widely distributed lineage. This lineage is characterized by substantial genomic diversification, long-term persistence, and transcontinental dissemination. Moreover, lineage IV subclades are distinguished by clade-defining SNP signatures, with the L gene containing the largest absolute number of lineage-defining variants. Codon-based analyses further showed that all six major PPRV genes were predominantly governed by purifying selection. These findings advance the understanding of PPRV evolution and provide an integrated phylogenetic, genomic, and selection-pressure framework for enhanced surveillance, outbreak investigations, and evidence-based interventions supporting the FAO-WOAH Global PPR Eradication Programme. However, integrating epidemiological information, such as livestock movement patterns, animal trade networks, vaccination history, and outbreak locations, with whole-genome sequencing of samples from underrepresented endemic regions and functional characterization of lineage-specific mutations will be essential to determine their biological significance and further strengthen global PPRV control and eradication efforts.

## Data Availability

The original contributions presented in the study are included in the article/Supplementary material, further inquiries can be directed to the corresponding authors.
