# Supplementary material for: Global evolutionary dynamics of peste des petits ruminants virus reveals geographic lineage structuring and clade-defining mutational signatures
Source: Front Microbiol. 2026 Sep 30;17:1944163. doi: 10.3389/fmicb.2026.1944163 (PMC13662165; doi:10.3389/fmicb.2026.1944163)
Supplement: Supplementary file 1 [file Image_1.doc]

Supplementary figure 1: Root to tip regression analysis of 212 WGSs of PPRV with a coefficient of determination (R²) of 0.8084.

Supplementary figure 2: Determination of mean evolutionary rate using Tracer with all the parameters having ESS value over 200
