# Supplementary material for: Global evolutionary dynamics of peste des petits ruminants virus reveals geographic lineage structuring and clade-defining mutational signatures
Source: Front Microbiol. 2026 Sep 30;17:1944163. doi: 10.3389/fmicb.2026.1944163 (PMC13662165; doi:10.3389/fmicb.2026.1944163)
Supplement: Supplementary file 2 [file Table_1.doc]

**Supplementary table 1A. Homoplasy assessment of diagnostic SNP positions.**

| **Analysis** | **Diagnostic SNP positions assessed** | **Potential homoplastic sites detected** |
| --- | --- | --- |
| Parsimony-based homoplasy assessment | 125 | 0 |

**Supplementary table 1B. Dataset-specific diagnostic SNP concordance threshold evaluation.**

| **Threshold** | **Correctly assigned genomes** | **Correct assignment rate (%)** | **Competing-clade false acceptance** |
| --- | --- | --- | --- |
| 0.50 | 151/152 | 99.34% | 2 genomes (1.32%) |
| 0.51 | 150/152 | 98.68% | 0 genomes (0%) |
| ≥0.51 | 150/152 | 98.68% | 0 genomes (0%) |

**Supplementary table 1C. Leave-one-out cross-validation performance of SNP-based subclade assignment.**

| **Clade** | **TP** | **FN** | **FP** | **Sensitivity** | **Specificity** | **Precision** | **F1** |
| --- | --- | --- | --- | --- | --- | --- | --- |
| A | 3 | 0 | 0 | 1.000 | 1.000 | 1.000 | 1.000 |
| B.1 | 23 | 0 | 0 | 1.000 | 1.000 | 1.000 | 1.000 |
| B.2a | 15 | 0 | 0 | 1.000 | 1.000 | 1.000 | 1.000 |
| B.2b | 110 | 1 | 0 | 0.991 | 1.000 | 1.000 | 0.995 |

Note: Threshold analysis indicates that a concordance threshold of 0.51 eliminated competing-clade acceptance within the evaluated dataset. LOOCV results represent internal validation using the available 152 Lineage IV genomes and should not be interpreted as external validation.
