# Supplementary material for: Global evolutionary dynamics of peste des petits ruminants virus reveals geographic lineage structuring and clade-defining mutational signatures
Source: Front Microbiol. 2026 Sep 30;17:1944163. doi: 10.3389/fmicb.2026.1944163 (PMC13662165; doi:10.3389/fmicb.2026.1944163)
Supplement: Supplementary file 3 [file Table_2.doc]

**Supplementary table 2.** Concordance between N- and F-gene phylogenetic classification and whole-genome-defined PPRV Lineage IV subclades.

| **Gene** | **Subclade** | **Whole-genome n** | **Members recovered in best-matching gene-tree clade** | **Exact recovery** | **F1 score** | **SH-aLRT / UFBoot** |
| --- | --- | --- | --- | --- | --- | --- |
| N | A | 3 | 3/3 | Yes | 1.000 | 100 / 100 |
| N | B.1 | 23 | 23/23 | Yes | 1.000 | 95 / 100 |
| N | B.2a | 15 | 15/15 | Yes | 1.000 | 96.6 / 100 |
| N | B.2b | 111 | 102/111 | No | 0.958 | 91.7 / 98 |
| F | A | 3 | 3/3 | Yes | 1.000 | 100 / 100 |
| F | B.1 | 23 | 22/23 | No | 0.978 | 51.8 / 31 |
| F | B.2a | 15 | 15/15 | Yes | 1.000 | 85.8 / 97 |
| F | B.2b | 111 | 105/111 | No | 0.972 | 82.3 / 59 |

**Note:** Overall macro-F1 was 0.989 for the N-gene tree and 0.988 for the F-gene tree. Nine diagnostic SNPs were identified within the N gene and 19 within the F gene; all were associated with subclade A in the present dataset.
